# Curcumin-Loaded Microemulsion Gel: An Optimized and Rheologically Acceptable Formulation with Conducive Dermatokinetics for Topical Breast Cancer Therapy

**DOI:** 10.3390/pharmaceutics18070897

**Published:** 2026-07-21

**Authors:** Md. Abul Barkat, Shakilur Rahman, Harshita Barkat, Abdulkareem A. Alanezi, Nader I. Namazi, Afaf F. Almuqati, Abrar Turki, Zahraa Alali, Mahesh Kumar Sharma, Kaisar Raza

**Affiliations:** 1 Department of Pharmaceutics, College of Pharmacy, University of Hafr Al Batin, Al Jamiah, Hafr Al Batin 39524, Saudi Arabia; 2 Department of Biology, College of Science, Imam Mohammad Ibn Saud Islamic University (IMSIU), Riyadh 11623, Saudi Arabia; 3 Department of Pharmaceutics and Pharmaceutical Industries, College of Pharmacy, Taibah University, Madinah 42353, Saudi Arabia; 4 Department of Pharmaceutical Chemistry, College of Pharmacy, University of Hafr Al Batin, Al Jamiah, Hafr Al Batin 39524, Saudi Arabia; 5 Clinical Nutrition Department, College of Applied Medical Sciences, University of Hafr Al Batin, Hafr Al Batin 39524, Saudi Arabia; 6 Department of Clinical Laboratory, College of Applied Medical Sciences, University of Hafr Al Batin, Hafr Al Batin 31991, Saudi Arabia; 7 ICFAI School of Pharmaceutical Sciences, The ICFAI University, Jaipur, Agra Road, Jamdoli, Jaipur 302031, Rajasthan, India; 8 Department of Pharmacy, Central University of Rajasthan, Bandarsindri, Ajmer 305817, Rajasthan, India

**Keywords:** breast cancer, curcumin, microemulsion, Carbopol gel, MCF7 cell line

## Abstract

**Background/Objectives**: Breast cancer remains one of the most prevalent malignancies worldwide, highlighting the need for safer and more effective therapeutic strategies. Curcumin has shown considerable anticancer potential; however, its clinical application is limited by poor aqueous solubility and low skin permeability. **Methods**: Therefore, a curcumin-loaded microemulsion (CUR-ME) was developed and optimized using a Box–Behnken design, followed by incorporation into a Carbopol 934 gel for topical breast cancer therapy. **Results**: The optimized formulation exhibited a particle size of 177.4 nm, a polydispersity index of 0.1309, and a zeta potential of −4.45 mV, indicating favorable physicochemical characteristics. CUR-ME demonstrated superior dose-dependent cytotoxicity against MCF-7 breast cancer cells, with a lower IC_50_ 8.89 µg/mL than free curcumin IC_50_ 9.83 µg/mL. Furthermore, Hoechst 33342 staining and the DCFDA assay confirmed enhanced apoptosis and intracellular reactive oxygen species generation in CUR-ME-treated cells, indicating improved cellular uptake and anticancer activity. Rheological and texture profile analyses demonstrated suitable viscosity, firmness, and spreadability for topical application. In vitro drug release revealed sustained release from the CUR-ME gel, achieved through the polymeric gel matrix, which increased viscosity, entrapped microemulsion droplets, and acted as a diffusion barrier to prolong drug retention and release. Confocal laser scanning microscopy further confirmed enhanced skin penetration of CUR-ME. **Conclusions**: Collectively, these findings demonstrate that the developed CUR-ME gel is a promising topical drug delivery system with sustained release, improved skin retention, and enhanced therapeutic potential for breast cancer management.

## 1. Introduction

Breast cancer remains one of the most commonly diagnosed malignancies and a major contributor to cancer-related deaths among women worldwide and is influenced by hormonal, lifestyle, environmental, and genetic factors. The risk increases substantially post-menopause, with nearly half of all cases occurring in women aged 50–69 years. In many developed regions, approximately 13% of women (1 in 8) may be diagnosed with breast cancer [1]. Although significant advancements have been made in early detection and treatment strategies, conventional modalities such as radiotherapy, chemotherapy, and hormonal therapy continue to face notable limitations. These include lack of specificity, systemic toxicity, development of drug resistance, and a range of undesirable side effects. Such challenges underscore the pressing demand for more effective, safer, and targeted therapeutic options. In this context, naturally occurring bioactive compounds have emerged as attractive candidates, owing to their ability to act on multiple targeted anticancer potentials and relatively lower toxicity profiles [2].

Curcumin (diferuloylmethane), a hydrophobic polyphenolic compound derived from *Curcuma longa* (turmeric), has attracted considerable attention because of its potent anti-inflammatory, antioxidant, and anticancer properties [3]. In breast cancer, particularly in MCF-7 cells, curcumin inhibits cell proliferation, induces apoptosis, increases intracellular ROS generation, and modulates multiple signaling pathways, including NF-κB, MAPK, Akt, and cyclin D1. Additionally, it suppresses tumor growth, angiogenesis, invasion, and metastasis, highlighting its potential as a promising chemopreventive and therapeutic agent for breast cancer treatment [4].

Breast cancer remains a major global health challenge despite significant advances in diagnosis and treatment. Although curcumin possesses well-documented anticancer, antioxidant, and anti-inflammatory properties, its clinical application for topical breast cancer therapy is severely limited by poor aqueous solubility, low skin permeability, rapid degradation, and poor bioavailability. Conventional topical formulations often fail to deliver sufficient drug concentrations to deeper skin layers while maintaining sustained therapeutic levels. Therefore, there is a need for an optimized topical delivery system capable of enhancing curcumin solubility, skin penetration, local retention, and controlled drug release [5].

To overcome these constraints, various formulation strategies have been examined, including polymeric nanoparticles, liposomes, solid lipid nanoparticles, and metallic NPs. Among these, microemulsions have appeared as an effective drug delivery approach owing to their distinctive physicochemical properties. Microemulsions are thermodynamically stable, isotropic systems containing oil, water, surfactant, and co-surfactant, with droplet sizes typically below 200 nm [6,7]. Their nanoscale structure enhances drug solubilization, improves permeability across biological membranes, and protects labile compounds like curcumin from degradation. Furthermore, microemulsions can significantly enhance drug absorption and cellular uptake, thereby improving therapeutic efficacy [8].

Despite these advantages, conventional microemulsions often suffer from low viscosity, which limits their retention at the site of application, particularly in topical delivery systems. To address this issue, microemulsion-based gels (ME gels) have been developed by combining Carbopol 934 as a gelling compound into the microemulsion system. These hybrid systems combine the superior solubilization and penetration-enhancing properties of microemulsions with the desirable rheological characteristics of gels, such as improved viscosity, spreadability, and prolonged residence time on the skin. This characteristic makes them particularly well-suited for localized drug-delivery applications [9].

Topical delivery of anticancer agents offers several advantages, including non-invasive administration, targeted drug localization, reduced systemic exposure, and improved patient compliance. In the case of breast cancer, localized delivery through the skin can enhance drug concentration at the tumor region while minimizing systemic adverse effects. The nanoscale droplets of microemulsions facilitate deeper penetration into the skin layers, thereby improving drug bioavailability and therapeutic outcomes [10].

The novelty of the present work lies in the development of a Box–Behnken Design-optimized curcumin-loaded microemulsion incorporated into a Carbopol 934 gel for topical breast cancer therapy. In addition to physicochemical optimization, the formulation was comprehensively evaluated through rheological and texture analyses, in vitro release kinetics, confocal laser scanning microscopy, cytotoxicity, Hoechst 33342 apoptosis staining, and DCFDA-based ROS generation assays, demonstrating enhanced skin penetration, sustained release, and improved anticancer activity compared with free curcumin.

The primary objective of the present work was to develop and optimize a curcumin-loaded microemulsion-based gel (CUR-ME gel) for enhanced topical breast cancer therapy with improved physicochemical stability, skin penetration, sustained drug release, and anticancer efficacy. Initially, a curcumin-loaded microemulsion was developed using appropriate oil, surfactant, and co-surfactant systems and optimized through a Box–Behnken Design (BBD) to evaluate the influence of formulation variables on particle size, polydispersity index (PDI), and zeta potential. The optimized formulation was further characterized for droplet morphology using transmission electron microscopy (TEM). Its in vitro anticancer potential was assessed in MCF-7 breast cancer cells using the MTT assay, while Hoechst 33342 staining and the DCFDA assay were performed to evaluate apoptosis and intracellular reactive oxygen species (ROS) generation, respectively.

Subsequently, the optimized microemulsion was incorporated into a Carbopol 934 gel to improve topical applicability and prolong drug retention. The CUR-ME gel was characterized for pH, spreadability, viscosity, texture profile, and rheological behavior. In vitro drug release studies and kinetic modeling were performed to evaluate the release mechanism, whereas confocal laser scanning microscopy (CLSM) was used to investigate skin penetration. The polymeric gel matrix was designed to provide sustained drug release by entrapping the microemulsion droplets, increasing viscosity, and acting as a diffusion barrier, thereby enhancing topical retention and therapeutic performance for breast cancer management.

## 2. Materials and Methods

### 2.1. Materials

Curcumin was procured from Sigma-Aldrich (Mumbai, India). Captex 300, Capmul MCM C8, Captex 355, Cremophor ELP, Capryol^®^ 90, and Transcutol^®^ P were provided by Gattefossé (Mumbai, India). Carbopol^®^ 934 and Tween 80 were supplied by CDH Fine Chemicals (New Delhi, India), while soybean and coconut oils were procured from Loba Chemie Pvt. Ltd. (Mumbai, India). Methanol, Formaldehyde, Rhodamine B dye, and triethanolamine were also obtained from CDH Fine Chemicals (New Delhi, India). The cell line MCF-7 (Cellosaurus CVCL_0031) was procured from the National Centre of Cell Culture (NCCS), Pune, Maharashtra, India, and Manipal University, Jaipur, India, and the revived cells were maintained at the Central University of Rajasthan, Rajasthan, India. All chemicals and reagents used in the research were of analytical grade.

### 2.2. Preparation of the Calibration Curve of Curcumin

An accurately weighed amount of curcumin (2 mg) was dissolved in methanol to obtain a stock solution (1000 µg/mL). A working solution of 5 µg/mL was prepared from the stock solution and scanned over the wavelength range of 200–800 nm using a UV–Visible spectrophotometer, with methanol as the blank. For calibration curve construction, appropriate dilutions of the stock solution were prepared in methanol to obtain concentrations of 1, 2, 3, 4, 5 and 6 µg/mL. The absorbance of each solution was measured at the finding wavelength, and the calibration curve was plotted with concentration (µg/mL) on the *x*-axis and absorbance on the *y*-axis. Each concentration was prepared in triplicate, and the absorbance values were recorded as the mean of three measurements to ensure accuracy and reproducibility (*n* = 3). A linear relationship between curcumin concentration and absorbance was obtained over the selected concentration range.

### 2.3. Selection and Screening of Excipients

#### 2.3.1. Identification of Suitable Oil

The solubility of curcumin (CUR), a lipophilic compound, was determined in various oils, including soybean oil, Captex 300, Capmul MCM C8, Captex 350, Coconut Oil, Castor oil, and Capryol^®^ 90, using a saturation shake-flask technique. Briefly, an excess amount of CUR was added to sealed glass vials containing each oil to determine its solubility. The mixtures were continuously agitated in an orbital shaker with temperature control that was kept at 37 ± 0.5 °C for 48 h to ensure complete saturation. After equilibration, the samples were allowed to settle, and then the undissolved drug was separated by centrifuging at 6000 rpm for ten minutes. To measure the solubility of CUR in each oil phase, the clear supernatant was gently collected, filtered with 0.22 µm, appropriately diluted with methanol, and examined using a validated UV–visible spectrophotometric technique at the predefined λ_max_ of curcumin [11].

#### 2.3.2. Screening for Surfactant and Co-Surfactant

Emulsification performance served as the basis for surfactant screening and optical clarity using a titration and spectrophotometric assessment method. Briefly, 0.5 mL of the selected oil phase was mixed with 5% (*v*/*v*) of each surfactant, including Transcutol P, Labrasol, Tween 80, Cremophor ELP, Acconon MC8-2, and Labrafac, in separate glass vials. The mixtures were gently heated to 40 °C and homogenized using a probe sonicator for 3 min to obtain homogeneous blends. Thereafter, each combination was diluted in 10 mL of distilled water while being gently stirred to facilitate emulsion formation. The emulsions were allowed to settle at room temperature for two hours and then visually examined for phase separation, clarity, and uniformity [12]. Subsequently, using distilled water as the reference, the % transmittance of each appropriately diluted emulsion was estimated at 510 nm using a UV–visible spectrophotometer. The surfactant that exhibited the highest transparency and transmittance and formed a stable emulsion was identified as the optimal surfactant for the microemulsion development [13].

#### 2.3.3. Screening of Surfactant Mixture Ratios

The compatibility and optimal surfactant (S_mix_) ratio were assessed using an aqueous titration method combined with visual stability evaluation. Briefly, the selected surfactant and co-surfactant were combined in various weight ratios (1:1, 2:1, 3:1, 4:1, and 5:1) to prepare the S_mix_ [14]. A fixed volume of the chosen oil was then added to each S_mix_ ratio, and a magnetic stirrer was employed to gently mix all ratios at 800 rpm for 20 min to ensure uniformity. Each blend was gradually titrated with distilled water while being continuously stirred to monitor spontaneous emulsion formation. The resultant systems were permitted to equilibrate for 12 h at ambient temperature and were visually examined for clarity, homogeneity, and the lack of phase separation or precipitation. The clarity of the emulsions was quantitatively assessed by determining the % transmittance at 510 nm with a UV–visible spectrophotometer. All experiments were performed in triplicate to ensure the reproducibility and accuracy of the results [15].

### 2.4. Construction of Pseudo-Ternary Phase

The water titration technique was employed to construct pseudo-ternary phase diagrams, combined with visual observation to identify the microemulsion region. Based on initial solubility assessments, the oil phase was selected as Capmul MCM C8, the surfactant was Transcutol P, and the co-surfactant was Cremophor ELP, with the aqueous phase consisting of distilled water. Surfactant–co-surfactant mixtures (S_mix_) were prepared at predetermined weight ratios and combined with the oil phase in a range of proportions from 1:9 to 9:1 in tightly sealed glass vials. Each oil–S_mix_ combination was homogenized on a mechanical shaker at 150 rpm for 10 min to attain uniform dispersion.

Distilled water was subsequently added dropwise using a calibrated burette under constant magnetic stirring at ambient temperature. After each addition, the samples’ transparency, clarity, and without phase separation were assessed visually to identify microemulsion formation. The compositions corresponding to transparent and stable systems were recorded. Phase diagrams were plotted using Tri-plot Software, Version 4.1 (Todd Thompson, USA) by marking the microemulsion region within the triangular coordinates representing oil, S_mix_, and aqueous phase concentrations, thereby identifying the optimal formulation range [16,17].

### 2.5. Formulation Development

The Curcumin-loaded microemulsions were formulated using a spontaneous emulsification technique [18]. Initially, a predetermined quantity of curcumin was mixed in the selected oil phase with constant stirring to obtain complete solubilization. Subsequently, a surfactant–co-surfactant mixture (S_mix_) comprising Transcutol P and Cremophor ELP in a fixed 3:1 ratio was incorporated into the oil phase and stirred to obtain a clear and uniform mixture. This resulting oil–S_mix_ blend was gradually added to the water phase while maintaining magnetic stirring at 800 rpm. The gradual addition enabled the spontaneous formation of a transparent, homogeneous, and thermodynamically stable oil-in-water (o/w) curcumin-loaded microemulsion, indicating successful microemulsion formulation [19].

### 2.6. Formulation Optimization

Applying the Quality by Design (QbD) strategy, the curcumin microemulsion (CUR-ME) formulation was systematically optimized to achieve a robust and high-quality product. QbD provides a structured framework that emphasizes product quality through the understanding of key formulation and process variables and their effect on major responses. The CUR-ME formulation was optimized in this work using Box Behnken Design (BBD) applied by Design-Expert^®^ software (version 12, Stat-Ease Inc., Minneapolis, MN, USA) [20]. Three levels of evaluation were conducted for three independent variables: oil content, surfactant concentration, and stirring speed. Particle size, zeta potential, and polydispersity index (PDI) were the dependent responses. To evaluate factor interactions and quadratic effects, the experimental findings were statistically evaluated using three-dimensional response surface methods and analysis of variance (ANOVA). BBD was selected because it efficiently evaluates nonlinear relationships while requiring fewer experimental runs, enabling rapid and cost-effective optimization of the microemulsion formulation [21].

### 2.7. Optimized Formulation Characterization

#### 2.7.1. Zeta Potential and Particle Size Analysis

The physicochemical properties of the developed curcumin microemulsion (CUR-ME), including droplet size, polydispersity index (PDI), and zeta potential, were estimated utilizing a Zetasizer Nano-ZS (Malvern Instruments, Malvern, UK) employing electrophoretic light scattering methods and dynamic light scattering (DLS). The experiments were conducted at 25 °C with a fixed scattering angle of 90°. Prior to analysis, the formulation was diluted by dispersing 0.1 mL of the microemulsion in Milli-Q water or normal saline to obtain a total volume of 1 mL, ensuring appropriate scattering intensity. Disposable polystyrene cuvettes were used to measure droplet size and PDI, whereas zeta potential was assessed based on electrophoretic mobility using a folded capillary cell in zeta potential mode. All estimated data were carried out in triplicate, and the data were reported as mean and standard deviation [22].

#### 2.7.2. Morphological Examination

The curcumin microemulsion’s (CUR-ME) morphological properties were characterized using transmission electron microscopy (TEM). The analysis was carried out using a TEM instrument (Tecnai G2 S-Twin, Eindhoven, Netherlands/Thermo Fisher Scientific Talos L120C, Waltham, MA, USA) operated at an accelerating voltage of 120 kV to obtain high-resolution images. Before imaging, the microemulsion was diluted in distilled water, and a drop of the diluted sample was put on a copper grid (300 mesh) covered with formvar and left to adsorb. After 30 s of negative staining with 1% phosphotungstic acid, the sample was cleaned with deionized water and allowed to dry naturally at room temperature. The dried grids were examined under TEM at appropriate magnification, and image analysis was performed using dedicated imaging software [23].

### 2.8. Cell Line Studies

#### 2.8.1. Cell Line-Based In Vitro Cytotoxicity Evaluation

The cytotoxic activity of curcumin (CUR) and curcumin microemulsion (CUR-ME) was assessed using the MTT test in the MCF-7 breast cancer cell line. Briefly, MCF-7 cells were inoculated into 96-well plates at a density of 10 × 10^3^ cells per well and incubated for 24 h at 37 °C in an atmosphere that was humidified with 5% CO_2_ to stimulate cell attachment. After incubation, the medium was replaced with fresh medium containing different concentrations of CUR and CUR-ME (5–40 µg/mL), followed by further incubation for 24 and 48 h [24]. Post-treatment, the cells were gently washed with phosphate-buffered saline (PBS, pH 7.4), and 20 µL of MTT solution (5 mg/mL) was added to each well. The plates were incubated at 37 °C for 3–4 h under dark conditions to facilitate the formation of formazan crystals. After incubation, the culture medium was carefully aspirated, and 200 µL of dimethyl sulfoxide (DMSO) was added to solubilize the formed crystals. The optical density was then recorded at 570 nm using a microplate reader. All MTT tests were performed in triplicate, and the percentage of cell viability along with IC_50_ values was calculated from the corresponding dose–response curves [25].

#### 2.8.2. Hoechst 33342 Fluorescence Staining

Nuclear morphological alterations linked to apoptosis were assessed via Hoechst 33342 staining in MCF-7 human breast cancer cells. The cells were inoculated on 6-well plates and permitted to adhere overnight under usual culture conditions (37 °C, 5% CO_2_). Subsequent to attachment, the cells were administered the corresponding IC_50_ concentrations of curcumin and microemulsion nanoparticles and cultured for 24 h. Subsequent to treatment, the cells were gently rinsed with phosphate-buffered saline (PBS) and subsequently stained with Hoechst dye (5 µg/mL) for approximately 15–20 min in the absence of light. Hoechst dye permeates the cell membrane and associates with DNA, facilitating the visualization of nuclear morphology. The fluorescence was observed in the Agilent BioTek Cytation 5 Cell Imaging Multimode Reader utilizing the DAPI filter.

#### 2.8.3. DCFDA Assay

The formation of intracellular reactive oxygen species (ROS) in MCF-7 cancer cells was assessed using the 2′,7′-dichlorofluoresin diacetate (DCFDA) assay. Approximately 1.5 × 10^5^ MCF-7 cells were inoculated into a 6-well plate and permitted to adhere overnight under usual culture conditions (37 °C, 5% CO_2_). The cells were subsequently treated with the corresponding IC50 values of curcumin and microemulsion nanoparticles and incubated for an additional 24 h. Subsequent to treatment, the culture medium was discarded, and the cells were rinsed with phosphate-buffered saline (PBS).

The cells were then treated with a 10µM DCFDA working solution, produced in serum-free medium, for approximately 30 min at 37 °C in darkness. DCFDA permeates the cells and is deacetylated by intracellular esterases to yield non-fluorescent DCFH, which is subsequently oxidized by reactive oxygen species (ROS) to generate the highly fluorescent molecule DCF. Following incubation, surplus dye was eliminated by rinsing with PBS twice. The fluorescence intensity was quantified using the Agilent BioTek Cytation 5 Cell Imaging Multimode Reader with the GFP filter. The elevation in fluorescence intensity signified augmented intracellular ROS production in treated cells relative to untreated control cells, as assessed using ImageJ software (version 1.50).

### 2.9. CUR-ME GEL Preparation

1% *w*/*v* of Carbopol 934 was employed as a gelling material to transform the optimized CUR-ME into a gel formulation. It is well recognized for its clear appearance, excellent cohesive properties, and ease of removal. Briefly, Carbopol 934 (1% *w*/*v*) was precisely measured and dispersed in distilled water under continuous stirring with a magnetic stirrer (Remi Motors Ltd., Mumbai, India) at 800–1200 rpm, followed by overnight swelling. The hydrated Carbopol dispersion was subsequently neutralized with 0.05% *w*/*w* triethanolamine to produce the gel base. Subsequently, the optimized CUR-ME (1 mg/mL) was gradually incorporated into the gel with constant stirring to ensure uniform distribution. Ultimately, 0.01% benzalkonium chloride was used as a preservative to complete the formulation [26].

### 2.10. Physicochemical Evaluation of Curcumin Gel

#### 2.10.1. Physical Properties, Homogeneity, and pH

Before further physical characterization, the CUR-ME gel was examined visually to assess its uniformity, absence of phase separation, and overall physical appearance. Color, transparency, and the presence of any visible particles or sediment were observed. Gel consistency and texture were assessed to determine whether the formulation exhibited a smooth gel-like structure without graininess. Spreadability was evaluated by applying the gel onto the skin surface. The pH was evaluated using a microprocessor pH meter (India). Every experiment was conducted at 25 ± 1 °C in triplicate.

#### 2.10.2. Rheology and Viscosity

The rheological behavior of the curcumin microemulsion gel (CUR-ME gel) was evaluated to determine its flow properties. Rheological properties were evaluated using a dynamic rheometer (MCR 102e, Anton Paar, Graz, Austria) fitted with a plate–plate configuration (PP-2-5) and coupled to a temperature-controlled circulating water system. The investigation was carried out at 25.0 ± 0.5 °C, and data acquisition was managed using Rheoplus software (RHEOPLUS/32 V3.21) [22]. The suitable amount of gel was placed on the plate, and the parameters were adjusted according to the instrument guidelines. Flow curve analysis was carried out to assess the viscosity and shear stress of the formulation. To characterize the flow behavior, Viscosity (Pa·s) and shear stress (τ) were assessed across a shear rate range of about 0.1–100 s^−1^. All analyses were repeated in triplicate [27].

#### 2.10.3. Analysis of Texture

The mechanical and textural characteristics of the developed curcumin microemulsion gel (CUR-ME gel) were assessed through texture profile analysis (TPA) utilizing a texture analyzer (TA.XT Plus, Stable Micro Systems, Godalming, UK). Approximately 100–120 mL of gel was transferred into a cylindrical sample holder and allowed to equilibrate to remove entrapped air bubbles prior to testing. A cylindrical probe was used to compress the gel sample under controlled conditions to simulate the deformation behavior of semisolid formulations. The pre-test, test, and post-test speeds were maintained at 1.5 mm/s, 2 mm/s, and 2 mm/s, respectively, while the trigger force was fixed at 10 g and the target distance at 20 mm. The force–time curves generated during compression were analyzed using the instrument software to determine key parameters such as gel strength, adhesiveness, cohesiveness, firmness, and springiness, which collectively describe the structural integrity and mechanical performance of the gel formulation [28].

### 2.11. Drug Release In Vitro Investigation

The in vitro drug release kinetics of curcumin (CUR) from CUR-ME and CUR-ME gel formulations were assessed using the dialysis bag diffusion method under sink conditions. Dialysis bags with a molecular weight cut-off of 10 kDa were utilized. Briefly, 2 mL of each formulation, CUR standard solution, CUR-ME, and CUR-ME incorporated into Carbopol gel, was loaded into the dialysis bag and securely sealed at both ends. The prepared bags were then immersed in 100 mL phosphate-buffered saline (PBS, pH 7.4), which served as the release medium. The experiment was performed at 37 ± 2 °C with constant stirring at 100 rpm to simulate physiological conditions and ensure uniform drug diffusion. At specified time points (0.5, 1, 2, 4, 6, 8, and 24 h), 2 mL of the release medium was withdrawn and immediately replenished with an equal volume of fresh PBS to preserve sink conditions and maintain a constant volume. The collected aliquots were filtered with a 0.22 µm filter and analyzed using a UV–Visible spectrophotometer at 425 nm, with PBS used as the blank. All measurements were performed in triplicate (n = 3), and the results are expressed as mean ± standard deviation. In addition, the cumulative drug release data were subjected to kinetic analysis using various models, including zero-order, first-order, Higuchi, and Korsmeyer–Peppas models, to elucidate the release behavior. The model exhibiting the highest correlation coefficient (R^2^) was considered the best fit for the release kinetics [29,30].

### 2.12. Confocal Assessment

To assess the skin deposition profile of the formulations, Franz diffusion cells were employed. Rhodamine B dye was used as a fluorescent probe to visualize drug penetration and distribution within the skin. Rhodamine-loaded formulations, including a simple solution, microemulsion (ME), plain gel, and ME gel, were prepared following the same procedure used for CUR-ME and CUR-ME gel, with curcumin replaced by Rhodamine B dye. The excised skin of an albino rat was meticulously positioned between the donor and acceptor chambers of the Franz diffusion cell for the experiment. The acceptor compartment was filled with phosphate-buffered saline (PBS, pH 7.4), while the donor compartment contained the respective Rhodamine-loaded formulations (solution, ME, plain gel, and ME gel). The system was maintained under constant stirring and allowed to run overnight to facilitate diffusion [31].

After completion of the experiment, the skin samples were removed, washed, and sectioned into thin slices for microscopic analysis. The prepared skin sections were gently placed on glass slides and observed using a confocal laser scanning microscope (CLSM) (Leica, Wetzlar, Germany) equipped with a confocal imaging system and a true confocal scanner spectral detector (TCS SPE). Imaging was performed at excitation and emission wavelengths of 488 nm and 560 nm, respectively. The captured micrographs were subsequently processed and evaluated using LAS AF software (Version 3.3) to assess and compare the penetration depth and distribution profiles of the formulations across different skin layers [32].

### 2.13. Stability Studies

The stability study was performed to evaluate the physicochemical stability of the optimized CUR-ME formulation during storage. An accelerated stability study was conducted for three months in accordance with the ICH Q1A(R2) guidelines. The formulation was stored under accelerated conditions at 40 ± 2 °C and 75 ± 5% relative humidity (RH) to assess the effects of elevated temperature and humidity on its stability. Samples were withdrawn and analyzed at predetermined time points, including baseline (0 month) and after 1, 2, and 3 months of storage. The evaluated parameters included particle size, polydispersity index (PDI), and zeta potential.

### 2.14. Statistical Analysis

Statistical analysis of all experimental results was performed with GraphPad Prism (version 8.0, San Diego, CA, USA). Data are reported as mean ± SD or mean ± SEM, as suitable. Group comparisons were conducted using one-way ANOVA, and *p* ≤ 0.05 was regarded as statistically significant.

## 3. Results

### 3.1. Determination of λmax and Calibration Curve of Curcumin

**Figure 1:**
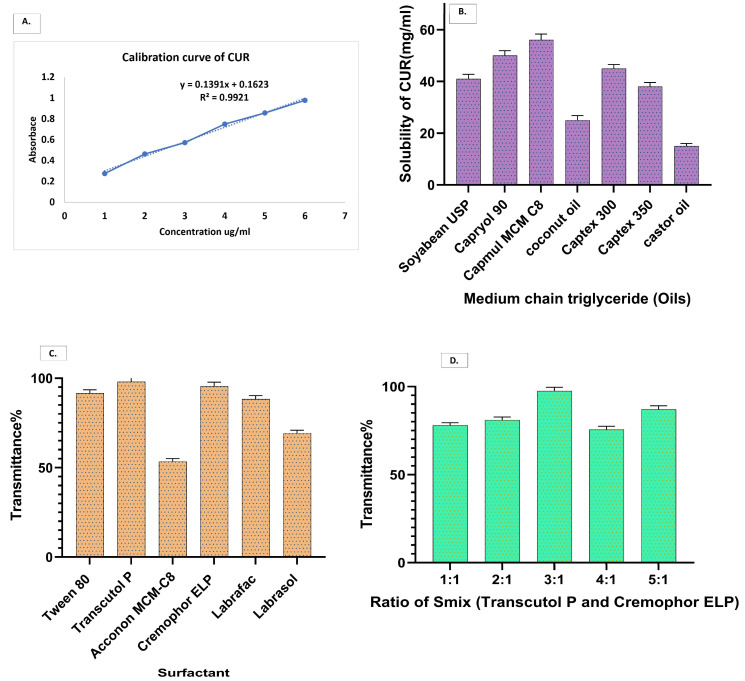
(**A**) Calibration curve of Curcumin in PBS (pH 7.4). (**B**) illustrates the solubility of Curcumin in oil (medium-chain triglycerides and long-chain triglycerides). (**C**) %Transmittance of the surfactants. (**D**) %Transmittance of the different ratios of S_mix_.

The UV–Visible spectrophotometric analysis showed that curcumin exhibited a maximum absorption wavelength (λmax) at 425 nm, which was selected for subsequent quantitative analysis. A calibration curve was constructed over the concentration range of 1–6 µg/mL, demonstrating a linear relationship between curcumin concentration and absorbance (Figure 1A). The regression equation was y = 0.1391x + 0.1623 with a correlation coefficient (R^2^ = 0.9921), indicating excellent linearity within the selected concentration range. The high correlation coefficient confirms the reliability, accuracy, and suitability of the developed UV–Visible spectrophotometric method for the quantitative estimation of curcumin in subsequent formulation characterization.

### 3.2. An Evaluation and Selection of Excipients

The oil phase was determined based on its ability to dissolve curcumin (CUR) and its appropriateness for microemulsion development. For solubility assessment, an excess quantity of CUR was separately introduced into different lipid vehicles, including soybean oil, Captex 300, soyabean oil, Captex 355, Capmul MCM C8, coconut oil, castor oil, and Capryol^®^ 90. The mixtures were initially vortexed for 10 min and subsequently maintained under constant agitation at room temperature for 72 h to reach equilibrium. Post-incubation, the samples were subjected to centrifugation at 5000 rpm for 30 min to separate insoluble drug particles. The transparent supernatant was carefully extracted, filtered with a 0.22 µm filter, appropriately diluted with methanol, and quantified spectrophotometrically at 425 nm. Among the screened oils, Capmul MCM C8 demonstrated the greatest solubilizing efficiency for CUR and was therefore selected as the oil phase for further development of the formulation, as presented in Figure 1B.

Surfactants and co-surfactants were assessed according to their effectiveness in emulsifying the selected oil phase, Capmul MCM C8. For this evaluation, equal proportions of oil and each surfactant were combined, and the mixtures were then diluted with distilled water while being gently stirred. The emulsification efficiency was evaluated by estimating the percentage transmittance of the resulting dispersions using a UV–visible spectrophotometer. Higher transmittance values indicated better emulsification and improved system clarity. Among the tested surfactants, the emulsification efficiency followed the order: Transcutol P > Cremophor ELP > Tween 80 > Acconon MC8-2 > Labrafac > Labrasol. Transcutol P showed the highest transmittance (97%), followed by Cremophor ELP (95%) (Figure 1C). Thus, Transcutol P and Cremophor ELP were employed as the surfactant and co-surfactant, respectively, for the development of the formulation.

Selected surfactant mixtures (S_mix_) were further optimized to determine the most effective ratio for microemulsion formation. Transcutol P and Cremophor ELP were combined in varying ratios of 1:1, 2:1, 3:1, 4:1, and 5:1, and each mixture was evaluated for its emulsification ability with Capmul MCM C8. The prepared mixtures were diluted with distilled water and analyzed for clarity through measurement of percent transmittance with a UV–visible spectrophotometer. Higher transmittance indicated improved emulsification efficiency and better system transparency. Among the tested ratios, the emulsification performance followed the order: 3:1 > 2:1 > 1:1 > 4:1 > 5:1. The corresponding transmittance values were 98.64%, 96.33%, 96.01%, 95.67%, and 93.56%, respectively (Figure 1D). Among the tested ratios, S_mix_ (3:1) exhibited maximum clarity and emulsification efficiency, leading to its selection for further development to obtain a stable formulation.

### 3.3. Preparation of Pseudo-Three-Component Phase Diagrams

Pseudo-ternary phase diagrams were constructed to identify the microemulsion region using Capmul MCM C8 as the oil phase, Transcutol P and Cremophor ELP as the surfactant/co-surfactant (Smix), and water as the aqueous phase. Sixteen oil-to-Smix ratios (1:9, 2:8, 3:7, 4:6, 5:5, 6:4, 7:3, 8:2, 9:1, 1:2, 1:3, 1:3.5, 1:5, 1:6, 1:7, and 1:8) were systematically evaluated by aqueous titration. Microemulsion formation was confirmed by visual assessment of transparency, homogeneity, and the absence of phase separation. Among the tested compositions, the 4:6 oil-to-Smix ratio produced the largest transparent microemulsion region, indicating the highest emulsification efficiency and widest compositional range for stable microemulsion formation (Figures S1 and S2). Ratios containing either lower or higher surfactant proportions exhibited comparatively smaller microemulsion regions and a greater tendency toward conventional emulsions. Based on these observations, the 4:6 oil-to-Smix ratio was selected for subsequent formulation optimization and development of the curcumin microemulsion.

### 3.4. Formulation Development

**Figure 2:**
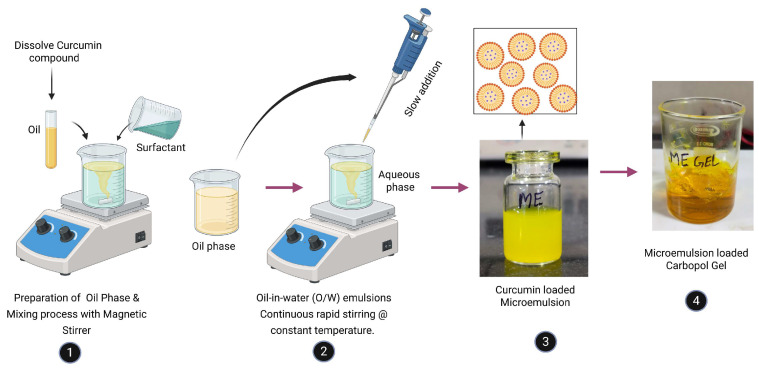
Preparation of Curcumin-loaded Microemulsion by the spontaneous emulsification method.

Curcumin-loaded microemulsions were formulated using the spontaneous emulsification technique. In brief, 5 mg of curcumin was solubilized in 120 μL of Capmul MCM C8 (oil phase) using a vortex mixer. A surfactant–co-surfactant mixture (S_mix_) consisting of Transcutol P and Cremophor ELP in a 3:1 ratio was then added and stirred to obtain a clear oil–S_mix_ mixture. The prepared mixture was then introduced slowly in a dropwise manner into the aqueous phase under constant magnetic stirring at 800 rpm, leading to the formation of a uniform oil-in-water microemulsion. The formation of a clear system indicated successful microemulsion development, as illustrated in Figure 2. Furthermore, A Quality by Design (QbD) framework was employed to systematically optimize formulation variables for enhanced stability and performance.

### 3.5. Formulation Optimization

A Quality by Design (QbD) strategy was implemented to optimize the CUR microemulsion formulation using Design Expert^®^ software (Version 12). A Box–Behnken Design (BBD) was utilized to systematically investigate the influence of three independent variables, namely oil concentration (A), S_mix_ ratio (B), and stirring speed (C). Their effects were evaluated on key quality attributes, including particle size (Response 1), polydispersity index (Response 2), and zeta potential (Response 3). The BBD model facilitated efficient optimization with a reduced number of experimental runs compared to traditional trial-and-error methods. A total of 17 experimental formulations were designed, prepared, and evaluated for their physicochemical characteristics, as summarized in Table 1. The generated data were analyzed statistically using analysis of variance (ANOVA) to determine the significance of the model and the interactions between variables. The findings revealed that the responses were best described by quadratic models, showing good conformity as revealed by the coefficient of variation (CV), standard deviation (SD), and determination coefficient (R^2^). The comprehensive statistical details of the developed models are presented in Table 2.

#### 3.5.1. Influence of Independent Factors on Particle Size

**Figure 3:**
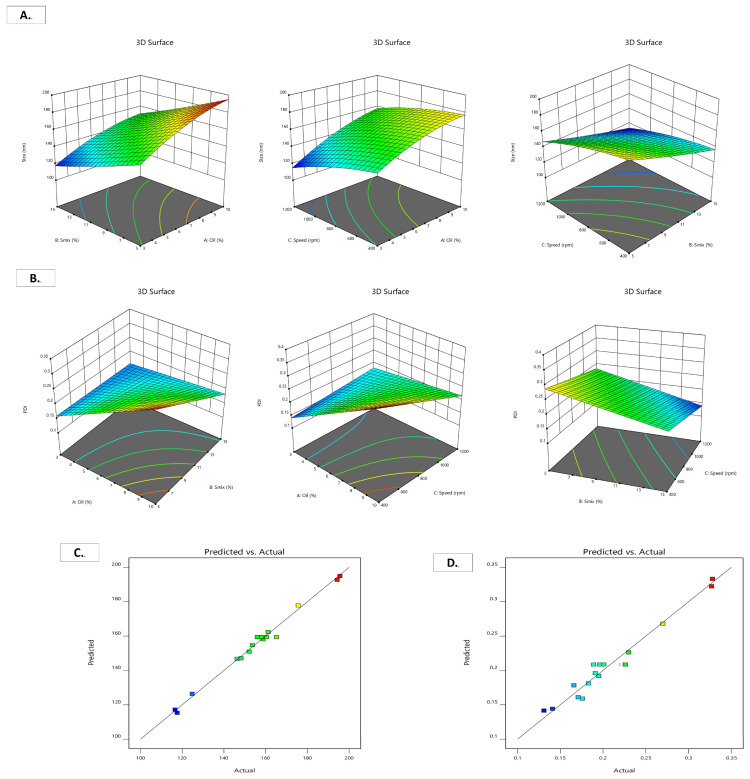
Effect of independent variables on dependent variables, such as (**A**) particle size and (**B**) PDI, whereas plots (**C**,**D**) depict the relationship between the actual and predicted R-square.

Particle size is a crucial parameter in microemulsion systems, as it directly influences drug release behavior, skin deposition, and permeation efficiency. For effective drug delivery, the particle size is typically maintained within the 100–200 nm range, with a mean particle size of approximately 150 nm considered optimal for balancing stability, safety, and therapeutic performance. A quadratic model, Equation (1), was constructed to depict the impact of formulation variables on particle size. Statistical analysis indicated a good model fit, with a predicted R^2^ value of 0.9447 closely matching the adjusted R^2^ value of 0.9783, demonstrating strong model reliability. Furthermore, the regression analysis produced a coefficient of determination (R^2^) of 0.9905, reflecting a high correlation between independent variables and the response, with both positive and negative effects observed on particle size. The correlation between the predicted and observed experimental values is presented in Figure 3C. Here, the coded factors A, B, and C representing oil concentration, S_mix_ concentration, and stirring speed, respectively, were derived using Equation (1)

Particle size (Y1) = 159.42, +18.49 × A, −20.38 × B, −12.76 × C, −1.63 × AB, +2.95 × AC, +2.43 × BC, −6.26 × A^2^, +1.31 × B^2^, −3.61 × C^2^ (1)

Particle size was significantly affected by the coded factors A, B, and C. Increasing the oil concentration (A) resulted in larger droplets, whereas elevated S_mix_ concentration (B) and stirring speed (C) decreased particle size. The extent of the coefficients indicates the strength of each variable’s influence; for instance, a positive coefficient for A suggests that increasing oil concentration increases particle size. These interactions between variables and particle size are illustrated in Figure 3A.

#### 3.5.2. Influence of Independent Factors on the PDI

The polydispersity index (PDI) values of the seventeen developed formulations ranged from 0.131 to 0.328, indicating varying degrees of particle-size distribution. The impact of key formulation variables, including oil concentration, S_mix_ concentration, and stirring speed, on these quality attributes was systematically evaluated. A two-factor interaction (2FI) model was applied to analyze the effects and interactions of these variables on PDI, as illustrated in Figure 3B.

PDI (Y2) = +0.2086, +0.0486 × A, −0.0384 × B −0.0234 × C, −0.0375 × AB, −0.0418 × AC, −0.0025 × BC(2)

The developed model for PDI showed strong statistical performance, with an R^2^ value of 0.9603, adjusted R^2^ of 0.9365, and predicted R^2^ of 0.8932, all within an acceptable difference of less than 0.2. This suggests that the model accounts for nearly 95% of the variation in PDI, reflecting strong predictive performance. The close alignment between the adjusted and predicted R^2^ values further supports the accuracy and robustness of the model.

Analysis of regression coefficients revealed that variable A (+0.0486) had a positive effect on PDI, whereas variables B (−0.0384) and C (−0.0234) showed negative influences. This indicates that increasing oil concentration tends to increase PDI, while higher Smix concentration and stirring speed contribute to reducing it. The agreement between predicted and experimental PDI values is presented in Figure 3D.

#### 3.5.3. Influence of Independent Factors on the Zeta Potential

**Figure 4:**
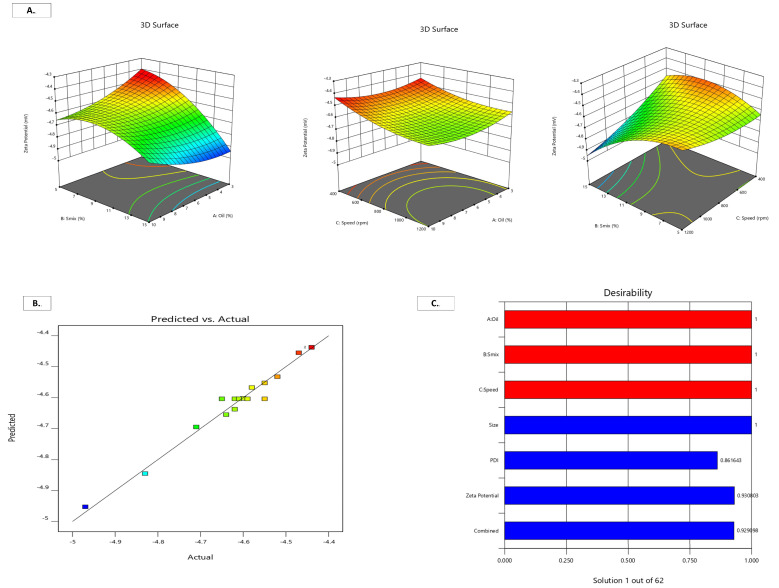
Effect of independent variables on dependent variables, such as (**A**) Zeta potential, whereas plots (**B**) depict the relationship between the actual and predicted R-square. (**C**) Overall desirability function plot showing the optimized curcumin-loaded microemulsion (CUR-ME) formulation predicted by the Box–Behnken Design.

The zeta potential values of the 17 developed formulations ranged between −4.44 and −4.97 mV. The influence of formulation variables, including oil concentration, S_mix_ concentration, and stirring speed, on this parameter was systematically evaluated (Figure 4A). A quadratic model was generated to describe the relationship between these variables and zeta potential, highlighting their individual and combined effects on the formulation.

Zeta potential (Y3) = −4.60, −0.0125 × A, −0.0875 × B, +0.0700 × C, +0.1075 × AB, −0.0125 × AC, −0.1225 × BC, +0.0533 × A^2^, −0.1117 × B^2^, +0432 × C^2^ (3)

The model demonstrated good agreement between the adjusted R^2^ (0.9408) and predicted R^2^ (0.8658), with a difference of less than 0.2, indicating reliable predictive performance. Regression coefficients showed that variables A (−0.0125) and B (−0.0875) had a negative impact on zeta potential, whereas variable C (+0.0700) exhibited a comparatively positive influence. The consistency between predicted and experimental values (Figure 4B) further confirms the accuracy of the model.

ANOVA results revealed that the developed models for particle size, PDI, and zeta potential were statistically significant, with non-significant lack-of-fit values, confirming the adequacy of the models. Based on these results, both numerical and graphical optimization approaches were employed to determine the optimal formulation region.

#### 3.5.4. Verification of the Optimized CUR-ME Formulation

The optimized CUR-ME formulation was established using the Box–Behnken Design (BBD) and desirability-function approach in Design-Expert^®^. The optimization criteria were set to minimize particle size (116.6–195.6 nm) and PDI (0.131–0.328), while maximizing zeta potential (−4.97 to −4.44 mV). Among 62 proposed solutions, the optimized formulation (Oil = 3%, Smix = 8.72348%, Speed = 800.004 rpm) exhibited a desirability value of 0.929, indicating excellent agreement between the predicted optimum and target responses, as shown in Table 3 and Figure 4C.

To validate the predictive capability of the BBD model, three independent confirmatory experiments (n = 3) were performed under the optimized conditions. The predicted values generated by the model were compared with the experimentally observed mean ± SD values. The predicted particle size of 152.27 nm closely matched the observed value of 151.4 ± 3.24 nm, with a prediction error of 0.57%. Similarly, the predicted PDI (0.1413) was in close agreement with the experimental value (0.130 ± 0.014), corresponding to a prediction error of 7.97%. The predicted zeta potential (−4.426 mV) was also consistent with the observed value (−4.487 ± 0.032 mV), showing a prediction error of 1.36%.

Furthermore, 95% prediction intervals (PI) were calculated for all responses to assess the reliability of the optimization model. The experimentally observed values for particle size (144.203–160.331 nm), PDI (0.1110–0.1715), and zeta potential (−4.507 to −4.345 mV) all fell within their respective 95% prediction intervals, confirming the statistical validity of the model, as shown in Table 4. These findings demonstrate that the desirability-function optimization was successfully validated experimentally and that the optimized formulation is robust, reproducible, and accurately predicted by the Box–Behnken Design model. The close agreement between predicted and experimental values, together with low prediction errors and inclusion within the 95% prediction intervals, confirms the reliability and predictive performance of the developed optimization model.

### 3.6. Optimized Formulation Characterization

**Figure 5:**
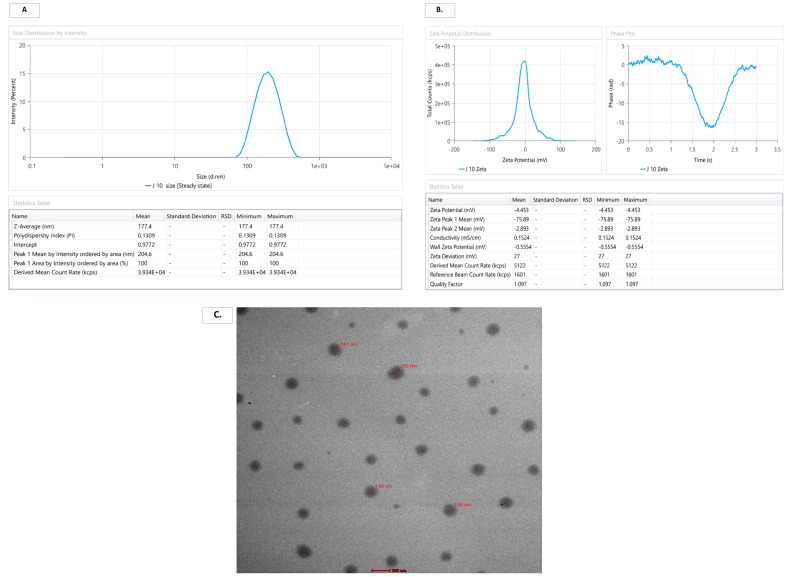
Figures (**A**–**C**) illustrate the characterization of the prepared curcumin-loaded microemulsion (ME), where (**A**) represents the particle size and polydispersity index (PDI) analysis, (**B**) shows the zeta potential profile, and (**C**) presents TEM micrographs for morphological evaluation, respectively.

The optimized CUR-ME formulation exhibited an average droplet size of approximately 177.4 nm with a low polydispersity index (PDI) of 0.1309, as analyzed via dynamic light scattering using a Zetasizer Nano-ZS (Malvern Instruments, UK) (Figure 5A). The low PDI indicates a uniform and monodisperse particle distribution without significant aggregation. This nanoscale size range supports efficient drug delivery and enhances skin permeation, as particles smaller than ~200 nm are known to penetrate the skin barrier more efficiently [33]. The optimized CUR-ME exhibited a zeta potential of −4.87 mV (Figure 5B), indicating adequate surface charge to maintain colloidal stability and reduce particle aggregation. This stability supports its suitability for transdermal delivery. Transmission electron microscopy further validated the particle size results, showing predominantly spherical particles below 190 nm. Overall, the formulation displayed particle sizes under 200 nm, suggesting effective skin penetration and potential accumulation at the target site, as illustrated in Figure 5C.

### 3.7. Cell Line Studies

#### 3.7.1. Cell Line-Based In Vitro Cytotoxicity Evaluation

**Figure 6:**
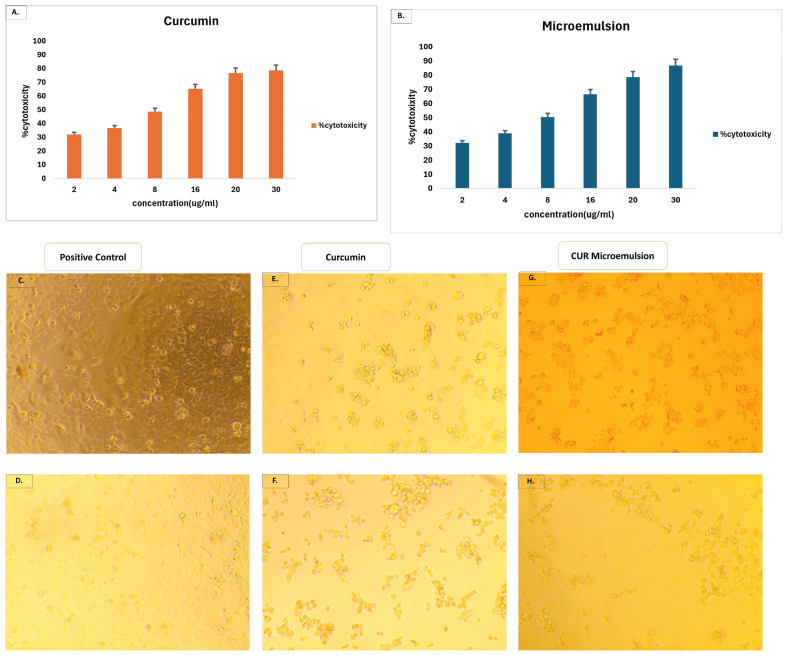
(**A**,**B**) Comparative Evaluation of % Cell Viability and Cytotoxic Effects of Curcumin and Curcumin-Loaded Microemulsion in MCF-7 Cell Lines. (**C**–**H**) Micrographs showing the synergistic cytotoxicity of the CUR and CUR Microemulsion treatment. (**C**,**D**) Positive control; (**E**,**F**) Curcumin; (**G**,**H**) Curcumin Microemulsion.

The cytotoxic potential of free curcumin (CUR) and curcumin-loaded microemulsion (CUR-ME) was evaluated in MCF-7 cells using the MTT assay after 24 h of treatment. Both formulations exhibited a concentration-dependent reduction in cell viability over the tested concentration range (2–30 µg/mL), confirming their dose-dependent antiproliferative activity. CUR-ME demonstrated slightly higher cytotoxic efficacy than free CUR, with an IC_50_ value of 8.89 µg/mL compared with 9.83 µg/mL for CUR. At higher concentrations, CUR-ME produced greater cytotoxicity (86.79% at 30 µg/mL) than free CUR (78.59% at 30 µg/mL), indicating enhanced anticancer activity. The improved cytotoxic effect of CUR-ME may be attributed to its nanosized droplets, which facilitate enhanced cellular uptake, improved intracellular drug delivery, and increased bioavailability of curcumin, resulting in more effective inhibition of MCF-7 cell proliferation (Figure 6A,B).

Furthermore, morphological assessment (Figure 6C–H) revealed significant cellular alterations in treated groups compared to untreated control cells. Cells treated with CUR and CUR-ME displayed typical signs of cytotoxicity, such as cell shrinkage, membrane blebbing, detachment from the substrate, and altered cellular morphology, suggesting induced cellular damage. Overall, these findings demonstrate that while CUR-ME retains anticancer activity, pure CUR exhibited comparatively greater cytotoxic efficacy in the MCF-7 cell model.

#### 3.7.2. Hoechst 33342 Fluorescence Staining

**Figure 7:**
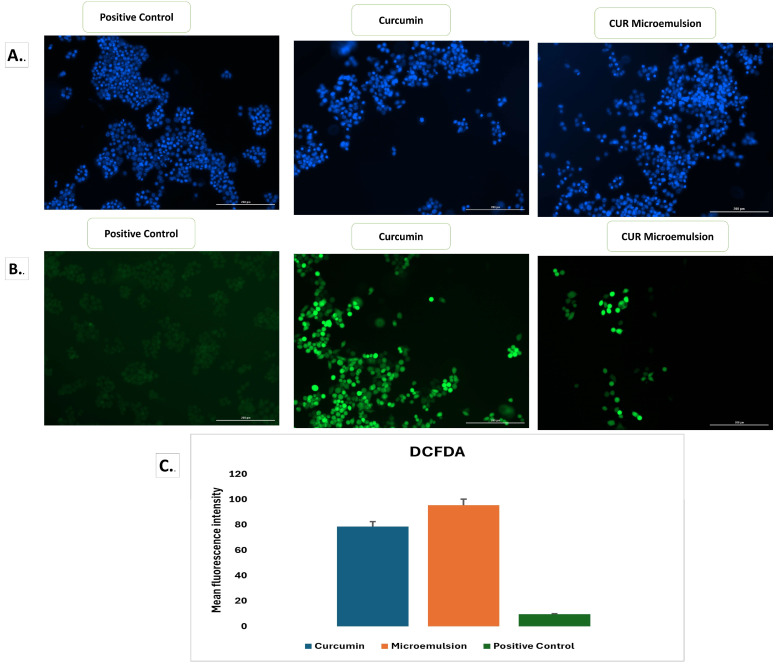
(**A**) Hoechst 33342 fluorescent staining of the nuclei in human breast MCF-7 cancer cells treated with Curcumin and CUR microemulsion for 24 h, where CUR microemulsion dose-treated MCF-7 cells showed the highest number of condensed nuclei compared to the IC50 of the CUR drug. Scale bar = 200 µm. (**B**) Cur- and CUR microemulsion elevation of intracellular ROS levels in MCF-7 cells. (**C**) Graphical analysis of mean DCFH-DA fluorescence in different dose-treated MCF-7 cells. Values were analyzed as mean ± SD of triplicates.

Hoechst 33342 staining was performed to evaluate apoptosis-induced nuclear morphological changes in MCF-7 cells following 24 h treatment with the IC_50_ concentrations of curcumin and CUR-loaded microemulsion. Untreated control cells exhibited intact, uniformly stained nuclei with normal morphology. In contrast, curcumin-treated cells displayed characteristic apoptotic features, including chromatin condensation, nuclear shrinkage, and partial nuclear fragmentation. These apoptotic changes were markedly more pronounced in cells treated with the CUR-loaded microemulsion, which exhibited intense nuclear condensation, extensive DNA fragmentation, and abundant apoptotic bodies, as shown in Figure 7A. The enhanced fluorescence intensity and severe nuclear damage observed in the microemulsion-treated group indicate greater induction of apoptosis compared with free curcumin, demonstrating improved intracellular delivery and anticancer efficacy of the optimized microemulsion formulation.

#### 3.7.3. ROS Generation in MCF-7 Cells by DCFDA Assay

Intracellular ROS generation in MCF-7 cells was evaluated using the DCFDA fluorescence assay following treatment with the IC_50_ concentrations of free curcumin and CUR-ME for 24 h. As shown in Figure 7B, untreated control cells exhibited minimal green fluorescence, indicating low basal ROS levels. In contrast, both treatment groups demonstrated a significant increase in intracellular ROS production, evidenced by enhanced DCF fluorescence intensity. Notably, CUR-ME-treated cells displayed markedly stronger fluorescence than free curcumin-treated cells, indicating greater ROS accumulation. Quantitative analysis using ImageJ confirmed the significantly higher fluorescence intensity in the CUR-ME group, as shown in Figure 7C. The elevated ROS levels induced by CUR-ME are likely to promote oxidative stress, resulting in mitochondrial dysfunction, DNA damage, and activation of apoptotic signaling pathways, thereby contributing to its enhanced cytotoxic and pro-apoptotic effects against MCF-7 cells.

### 3.8. Preparation and Evaluation of CUR Gel

The CUR-ME gel was prepared using 1% *w*/*v* Carbopol 934 due to its favorable cohesive properties and easy washability. Evaluation of the gel revealed a fine texture, uniform yellowish appearance, and absence of phase separation or cracking, confirming good consistency. The pH was measured at 6.54 ± 0.055 (25 ± 1 °C), indicating its suitability for dermal use. Additionally, the gel demonstrated satisfactory content uniformity, spreadability, and extrudability, along with favorable rheological behavior and texture profile. Overall, the absence of instability signs such as phase separation or structural breakdown confirms that the developed CUR-ME gel is stable, homogeneous, and appropriate for topical use.

### 3.9. Characterization of CUR Gel

#### 3.9.1. Rheology of CUR Gel

**Figure 8:**
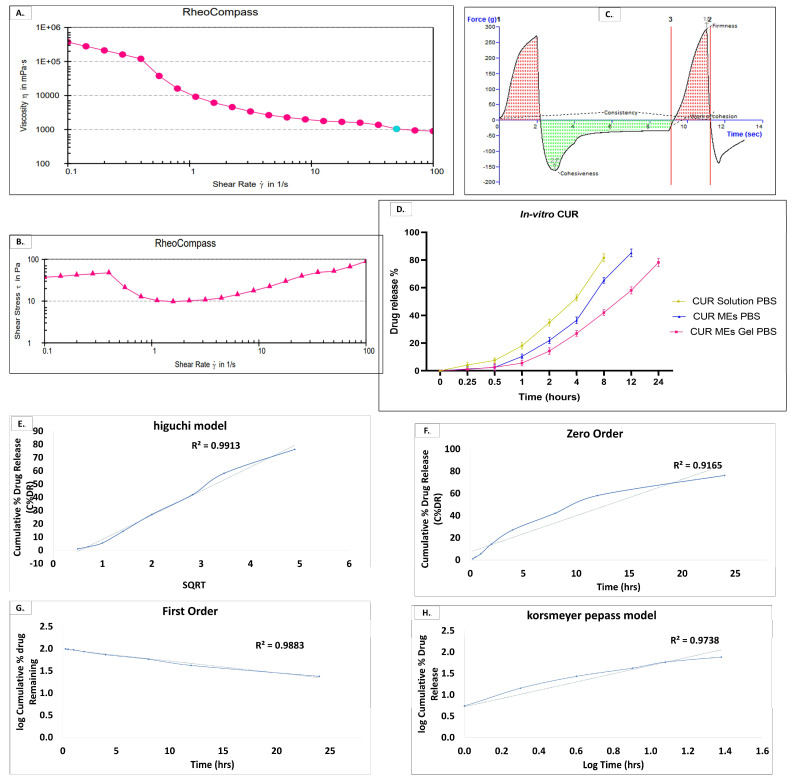
Illustration of the rheological behavior of optimized Curcumin microemulsion-loaded gel formulation. (**A**) Relationship between viscosity and shear rate of ME-loaded Carbopol gel. (**B**) Relationship between shear stress and shear rate of ME-loaded Carbopol gel. (**C**) Shows the texture profile analysis of CUR-ME gel. (**D**) In vitro release of CUR Solution, CUR-ME, and CUR-ME gel. (**E**–**H**) Drug release kinetic model of CUR-ME gel.

The rheological behavior of the optimized CUR-ME gel was evaluated using a rheometer to assess its suitability for topical breast cancer application. The flow properties were analyzed through rheograms depicting shear stress and viscosity across a wide shear rate range (0.099–100 s^−1^). The formulation exhibited a clear shear-thinning behavior, where the viscosity of the gel was found to be highly shear-dependent, confirming non-Newtonian flow behavior. Viscosity decreased markedly with increasing shear rate. Specifically, the viscosity reduced from approximately 3.71 × 10^5^ mPa·s at 0.099 s^−1^ to 898.4 mPa·s at 100 s^−1^, while shear stress increased progressively, indicating structured flow behavior. At a moderate shear rate of approximately 50 s^−1^, the viscosity measured around 1043 mPa·s, suggesting good spreadability and ease of application (Figure 8A,B). This reduction in viscosity under shear confirms that the gel becomes less resistant during application but regains viscosity afterward, ensuring prolonged residence at the skin site. Such pseudoplastic behavior, CUR gel, indicates strong internal network formation due to polymer entanglement and micellar interactions. Overall, the CUR-ME gel exhibits optimal rheological properties, supporting enhanced skin retention and effective localized drug delivery for breast cancer treatment.

#### 3.9.2. Texture Profiling of CUR-ME Gel

The mechanical behavior and spreadability attributes of the CUR-ME gel were analyzed using texture profile analysis, considering parameters such as firmness, consistency, work of adhesion, and cohesiveness. The force–time curve (Figure 8C) provided detailed insight into the gel’s deformation behavior and structural integrity. The firmness of the CUR-ME gel was found to be 294.12 g force, indicating a high resistance to deformation and confirming strong structural stability. Despite this high firmness, the gel exhibited satisfactory spreadability, suggesting an optimal balance between rigidity and ease of application, which is essential for uniform drug distribution on the skin in breast cancer therapy.

The consistency value was 231.22 g·s, reflecting moderate viscosity and a stable, homogeneous texture. This ensures that the gel maintains its form while enabling controlled drug release. Cohesiveness was recorded as −162.44 g force, with a work of cohesion of 291.43 g·s, indicating strong internal bonding and the ability to withstand repeated deformation. Additionally, the high work of adhesion suggests good stickiness, promoting prolonged skin contact. Overall, these properties indicate that the CUR-ME gel is well-suited for topical application, ensuring enhanced retention, sustained drug delivery, and improved therapeutic efficacy in breast cancer treatment.

### 3.10. In Vitro Release Kinetics Study

The epidermis and dermis represent the primary target tissues for topical breast cancer therapy; therefore, an ideal formulation should efficiently penetrate the stratum corneum while providing sustained drug release to maintain therapeutic concentrations within the skin and minimize systemic exposure. To evaluate the release characteristics, in vitro drug-release studies were performed using a dialysis bag for the CUR solution, CUR-ME, and CUR-ME gel. The cumulative drug release was found to be for the CUR solution (81.624 µg/cm^2^) at 8 h, followed by CUR-ME (85.156 µg/cm^2^) at 12 h, and for the CUR-ME gel (78.189 µg/cm^2^) at 24 h (Figure 8D). Although the CUR-ME gel exhibited a comparatively lower cumulative release, its gradual release profile indicates a sustained-release behavior that is advantageous for prolonged topical therapy.

The sustained release from the CUR-ME gel can be attributed to the three-dimensional gel network, which acts as an additional diffusion barrier surrounding the microemulsion droplets. This polymeric matrix restricts the mobility of both the encapsulated droplets and the dissolved drug molecules, thereby slowing drug diffusion into the release medium. Moreover, the increased viscosity of the gel enhances formulation retention at the application site, prolonging the residence time on the skin and facilitating continuous drug diffusion into deeper skin layers. Such controlled release is expected to maintain effective local drug concentrations while reducing the frequency of application and minimizing systemic absorption.

To further elucidate the release mechanism, the release data were fitted to different kinetic models. As shown in Figure 8E–H, among the tested models, the Higuchi model exhibited the highest correlation coefficient (R^2^ = 0.9913), indicating that drug release was predominantly governed by diffusion through the gel matrix. The excellent fit to the first-order (R^2^ = 0.9883) and Korsmeyer–Peppas (R^2^ = 0.9738) models further suggests that drug release is concentration-dependent and influenced by diffusion through the polymeric network. Collectively, these findings demonstrate that incorporation of the microemulsion into the gel matrix effectively modulates curcumin release, providing sustained drug delivery, prolonged skin retention, and improved suitability for topical breast cancer therapy.

### 3.11. In Vivo Confocal Study

**Figure 9:**
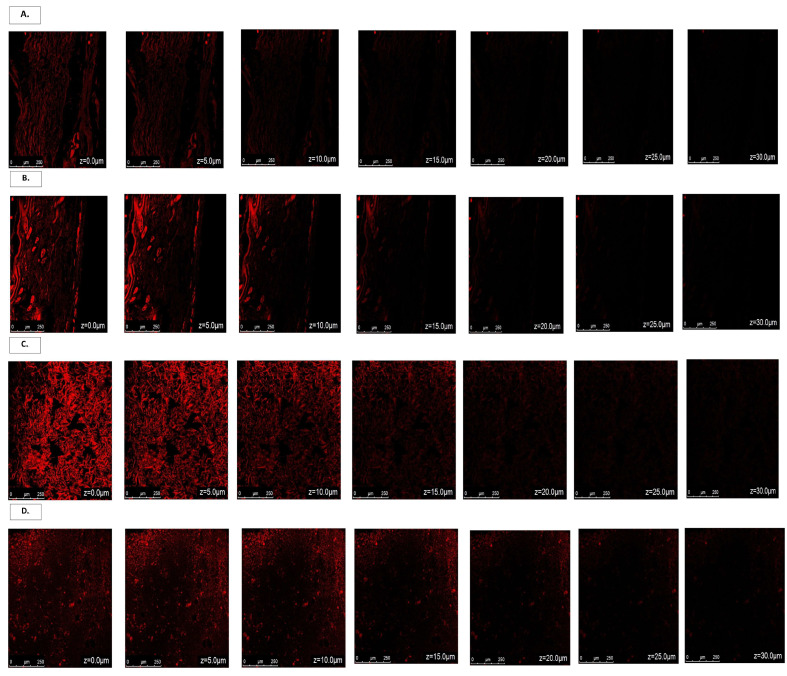
Microscopic depth analysis was performed using confocal laser scanning microscopy (CLSM). (**A**) Confocal images illustrate Rhodamine B dye deposition from the simple solution, (**B**) microemulsion, (**C**) plain gel, and (**D**) microemulsion gel, enabling comparative assessment of skin penetration and distribution.

Evaluation of drug deposition within skin layers is essential for ensuring effective topical delivery, as the therapeutic concentration must reach the epidermis and dermis to exert its action. In this study, skin penetration was assessed using Rhodamine-loaded formulations, including simple solution, microemulsion (ME), plain gel, and CUR-ME gel. Skin sections were prepared and analyzed using confocal laser scanning microscopy to observe fluorescence intensity at different depths (0–30 µm). At the surface 0 µm, all formulations showed maximum fluorescence intensity, which gradually decreased with increasing depth. The simple solution exhibited limited penetration, while the ME showed improved permeation due to its nanosized droplets. The plain gel demonstrated restricted diffusion; however, the CUR-ME gel displayed an enhanced, deeper fluorescence distribution across the epidermal and dermal layers, as shown in Figure 9.

The reduced intensity with depth confirms progressive permeation, while comparatively higher fluorescence in deeper layers for the CUR-ME gel indicates superior penetration ability. This enhanced deposition is likely due to the synergistic action of the microemulsion system and gel matrix, which facilitates sustained release and prolonged skin retention. Overall, the CUR-ME gel demonstrated efficient drug delivery to deeper skin layers, making it a promising system for topical breast cancer therapy.

### 3.12. Stability Studies

The stability of the optimized drug delivery system was assessed by monitoring key physicochemical parameters, including particle size, polydispersity index (PDI), and zeta potential, at monthly intervals throughout the storage period. No significant changes were observed in any of these parameters over the three-month study, indicating that the CUR-ME formulation maintained its physicochemical integrity and stability under accelerated storage conditions. The results of the accelerated stability study are summarized in Table 5.

## 4. Discussion

The present work successfully developed and optimized a curcumin-loaded microemulsion gel (CUR-ME gel) for topical breast cancer therapy using a systematic Box–Behnken Design (BBD) approach. The optimized formulation exhibited favorable physicochemical characteristics, enhanced biological activity, sustained drug release, and improved skin penetration, supporting its potential as an effective topical nanocarrier.

The BBD optimization identified the optimal formulation with a desirability value of 0.929. The optimized CUR-ME exhibited a particle size of 177.4 nm, PDI of 0.1309, and zeta potential of −4.45 mV, indicating a narrow size distribution and acceptable physical stability. Validation experiments showed close agreement between predicted and experimental responses with low prediction errors, confirming the robustness and predictive capability of the optimization model. The nanosized droplets provide a large surface area for drug solubilization and facilitate intimate contact with the skin, thereby enhancing topical drug delivery. Transmission electron microscopy confirmed spherical, uniformly distributed microemulsion droplets without aggregation, indicating successful formulation development. Complete solubilization of curcumin within the oil phase ensured homogeneous drug distribution and minimized the possibility of drug crystallization, thereby contributing to formulation stability and reproducible drug release.

The anticancer activity of the optimized formulation was evaluated in MCF-7 breast cancer cells using the MTT assay. Both free curcumin and CUR-ME exhibited concentration-dependent cytotoxicity; however, CUR-ME demonstrated greater efficacy, with a lower IC_50_ value (8.89 µg/mL) than free curcumin (9.83 µg/mL). At the highest tested concentration (30 µg/mL), CUR-ME produced 86.79% cytotoxicity, compared with 78.59% for free curcumin. The improved cytotoxicity can be attributed to enhanced drug solubilization, nanosized droplets that facilitate cellular uptake, and improved intracellular delivery of curcumin. The enhanced biological activity was further supported by Hoechst 33342 staining, which demonstrated significantly greater chromatin condensation, nuclear fragmentation, and apoptotic body formation in CUR-ME-treated cells than in free curcumin-treated cells. These findings indicate that incorporation into the microemulsion enhanced apoptosis induction. Similarly, the DCFDA assay revealed markedly higher intracellular ROS generation following CUR-ME treatment. Increased ROS levels promote oxidative stress, mitochondrial dysfunction, DNA damage, and activation of apoptotic signaling pathways, providing mechanistic evidence for the superior anticancer activity of CUR-ME.

To improve topical applicability, the optimized microemulsion was incorporated into a Carbopol 934 gel. The resulting CUR-ME gel exhibited an acceptable pH together with suitable viscosity, firmness, spreadability, and pseudoplastic (shear-thinning) behavior, making it appropriate for topical administration. The increased viscosity improves formulation retention at the application site while allowing easy spreading during application, thereby enhancing patient compliance and local drug availability. The in vitro release study demonstrated that incorporation of the optimized microemulsion into the Carbopol gel successfully provided sustained drug release. The polymeric gel network increased formulation viscosity, entrapped the microemulsion droplets, and acted as a diffusion barrier, thereby slowing drug diffusion and prolonging curcumin release. Release kinetic analysis showed the best fit with the Higuchi model, indicating that diffusion through the gel matrix was the predominant release mechanism. Such sustained release is advantageous for topical therapy because it prolongs drug residence at the target site while reducing dosing frequency. Confocal laser scanning microscopy demonstrated enhanced skin penetration of CUR-ME compared with the simple solution. The nanosized droplets and surfactant system facilitated deeper penetration into the skin layers, which is essential for achieving effective localized drug concentrations in topical breast cancer therapy.

Compared with previously reported curcumin topical nanocarriers, the developed CUR-ME gel combines the excellent solubilization and penetration-enhancing properties of microemulsions with the bioadhesive and sustained-release characteristics of a Carbopol gel. Unlike conventional nanoemulsions, which often exhibit poor retention because of low viscosity, the present formulation provides prolonged residence time while maintaining efficient drug delivery. Moreover, comprehensive evaluation of optimization, physicochemical properties, rheology, biological activity, apoptosis, ROS generation, and skin penetration highlights the superiority of the developed system. Overall, the optimized CUR-ME gel demonstrated improved physicochemical stability, enhanced cytotoxicity, greater apoptosis induction, increased ROS generation, sustained drug release, and enhanced skin penetration compared with free curcumin. These findings support its potential as a promising topical nanocarrier for localized breast cancer therapy. Future in vivo pharmacokinetic, biodistribution, efficacy, and dermal safety studies are warranted to further establish its translational potential for clinical application.

## 5. Conclusions

Curcumin-loaded microemulsion incorporated into a Carbopol 934 gel successfully addressed the limitations of curcumin by improving its topical delivery, physicochemical stability, and therapeutic performance. The optimized CUR-ME exhibited desirable particle size, narrow size distribution, and suitable zeta potential, confirming successful formulation and optimization. The formulation demonstrated enhanced cytotoxicity against MCF-7 breast cancer cells, with a lower IC_50_ than free curcumin, and its superior anticancer activity was further supported by increased apoptosis and intracellular ROS generation, as confirmed by Hoechst 33342 staining and the DCFDA assay. Rheological and texture profile analyses indicated appropriate viscosity, firmness, spreadability, and pseudoplastic behavior, making the gel suitable for topical application. The polymeric Carbopol gel matrix provided sustained drug release by entrapping the microemulsion droplets and acting as a diffusion barrier, while confocal laser scanning microscopy confirmed enhanced skin penetration. Collectively, these findings demonstrate that the developed CUR-ME gel is a promising topical nanocarrier with improved skin retention, controlled drug release, enhanced anticancer efficacy, and potential for localized breast cancer therapy. Further in vivo pharmacokinetic, efficacy, and safety studies are warranted to support its clinical translation.

## Figures and Tables

**Table 1:** The levels of various factors and their corresponding responses were analyzed to optimize the curcumin-loaded microemulsion using a Box–Behnken Design.

		Factor 1	Factor 2	Factor 3	Response 1	Response 2	Response 3
Std	Run	A: Oil	B: Smix	C: Speed	Size	PDI	Zeta Potential
		%	%	rpm	nm		mV
7	1	3	10	1200	117.6	0.166	−4.55
6	2	10	10	400	175.6	0.327	−4.44
10	3	6.5	15	400	148.2	0.191	−4.58
3	4	3	15	800	116.6	0.176	−4.83
9	5	6.5	5	400	194.3	0.27	−4.62
11	6	6.5	5	1200	161.2	0.23	−4.52
2	7	10	5	800	195.6	0.328	−4.71
8	8	10	10	1200	158.7	0.195	−4.6
16	9	6.5	10	800	158	0.226	−4.59
12	10	6.5	15	1200	124.8	0.141	−4.97
17	11	6.5	10	800	156	0.201	−4.65
5	12	3	10	400	146.3	0.131	−4.44
4	13	10	15	800	152.1	0.183	−4.64
14	14	6.5	10	800	160.4	0.196	−4.55
15	15	6.5	10	800	157.5	0.226	−4.61
1	16	3	5	800	153.6	0.171	−4.47
13	17	6.5	10	800	165.2	0.189	−4.62

**Table 2:** Regression analysis was performed to summarize the effects of independent variables A, B, and C on responses 1, 2, and 3 as part of the Quality by Design (QbD) analysis.

Quadratic Model	R^2^	Adjusted R^2^	Predicted R^2^	% CV	Adequate Precision
Response 1 Size	0.9905	0.9783	0.9447	2.08	32.05
Response 2 PDI	0.9603	0.9365	0.8932	6.74	21.24
Response 3 Potential	0.9741	0.9408	0.8658	0.7037	26.69

**Table 3:** Box–Behnken Design Optimization Constraints and Confirmation of the Optimized CUR-ME Formulation.

Constraints				Importance		Confirmation	Location #1
Name	Goal	Lower Limit	Upper Limit	3	Oil	Smix	Speed
A: Oil	is in range	3	10	3	3	8.72348	800
B: Smix	is in range	5	15	3		Response	data
C: Speed	is in range	400	1200	3	Size	PDI	Zeta Potential
Size	is in range	116.6	195.6	3	143.6	0.13	−4.49
PDI	minimize	0.131	0.328	3	154.4	0.15	−4.46
Zeta Potential	maximize	−4.97	−4.44		156.2	0.11	−4.51

**Table 4:** Validation of the Optimized CUR-ME Formulation Predicted by the Box–Behnken Design Using Confirmatory Experiments (n = 3).

Confirmation	Interval: Two-Sided	Confidence = 95%					
Solution 1 of 62 Responses Desirability (0.929)	Predicted Value Mean	Observed Value Mean	Std Dev	n	95% PI Low	95% PI High	Prediction Error (%)
Size	152.267	151.4	3.23862	3	144.203	160.331	0.57
PDI	0.141264	0.13	0.0140548	3	0.111017	0.171511	7.97
Zeta Potential	−4.42628	−4.48667	0.0324478	3	−4.50707	−4.34548	1.36

**Table 5:** Accelerated stability study of the optimized CUR-ME formulation under ICH Q1A(R2) conditions (40 ± 2 °C/75 ± 5% RH).

Storage Time (Months)	Particle Size (nm)	PDI	Zeta Potential (mV)
0 (Initial)	178.22	0.14	−4.05
1	181.12	0.19	−4.12
2	177.31	0.17	−4.71
3	176.76	0.15	−4.43

## Data Availability

The original contributions presented in the study are included in the article/Supplementary Materials; further inquiries can be directed to the corresponding authors.

## Supplementary Materials

The following supporting information can be downloaded at https://www.mdpi.com/article/10.3390/pharmaceutics18070897/s1. Figure S1: Pseudo-ternary phase diagrams were constructed using Capmul MCM-C8 as the oil phase, Transcutol-P as the surfactant, and Cremophor ELP as the co-surfactant. The squared region represents the O/W Microemulsion area observed at varying oil to Smix ratios: (a) 1:9, (b) 2:8, (c) 3:7, (d) 4:6, (e) 5:5, (f) 6:4, (g) 7:3, (h) 8:2. Figure S2: Pseudo-ternary phase diagrams were constructed using Capmul MCM-C8 as the oil phase, Transcutol-P as the surfactant, and Cremophor ELP as the co-surfactant. The squared region represents the O/W Microemulsion area observed at varying oil to Smix ratios: (a) 9:1, (b) 1:2, (c) 1:3, (d) 1:3.5, (e) 1:5, (f) 1:6, (g) 1:7, and (h) 1:8.
