# Supplementary material for: Curcumin-Loaded Microemulsion Gel: An Optimized and Rheologically Acceptable Formulation with Conducive Dermatokinetics for Topical Breast Cancer Therapy
Source: Pharmaceutics. 2026 Jul 21;18(7):897. doi: 10.3390/pharmaceutics18070897 (PMC13416279; doi:10.3390/pharmaceutics18070897)
Supplement: Supplementary file 1 [file pharmaceutics-18-00897-s001.zip › pharmaceutics-4385046-supplementary.pdf]

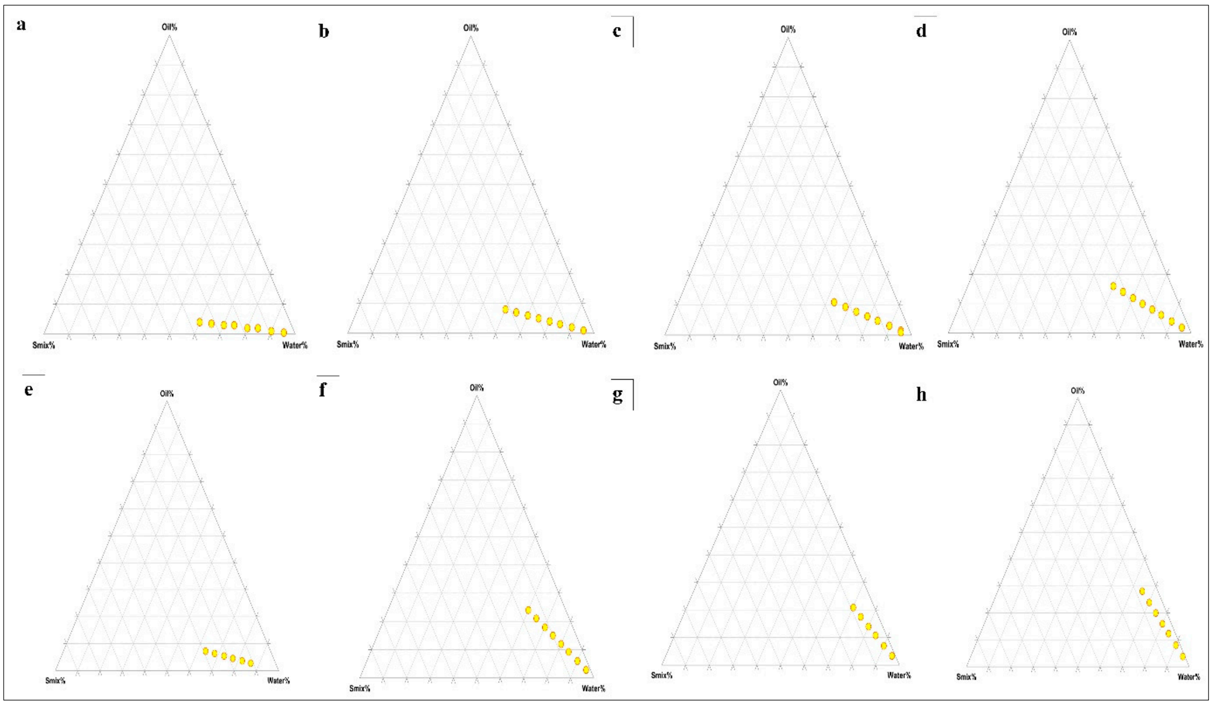

Figure S1: Pseudo-ternary phase diagrams were constructed using Capmul MCM-C8 as the oil phase, Transcutol-P as the surfactant, and Cremophor ELP as the co-surfactant. The squared region represents the O/W Microemulsion area observed at varying oil to Smix ratios: (a) 1:9, (b) 2:8, (c) 3:7, (d) 4:6, (e) 5:5, (f) 6:4, (g) 7:3, (h) 8:2.

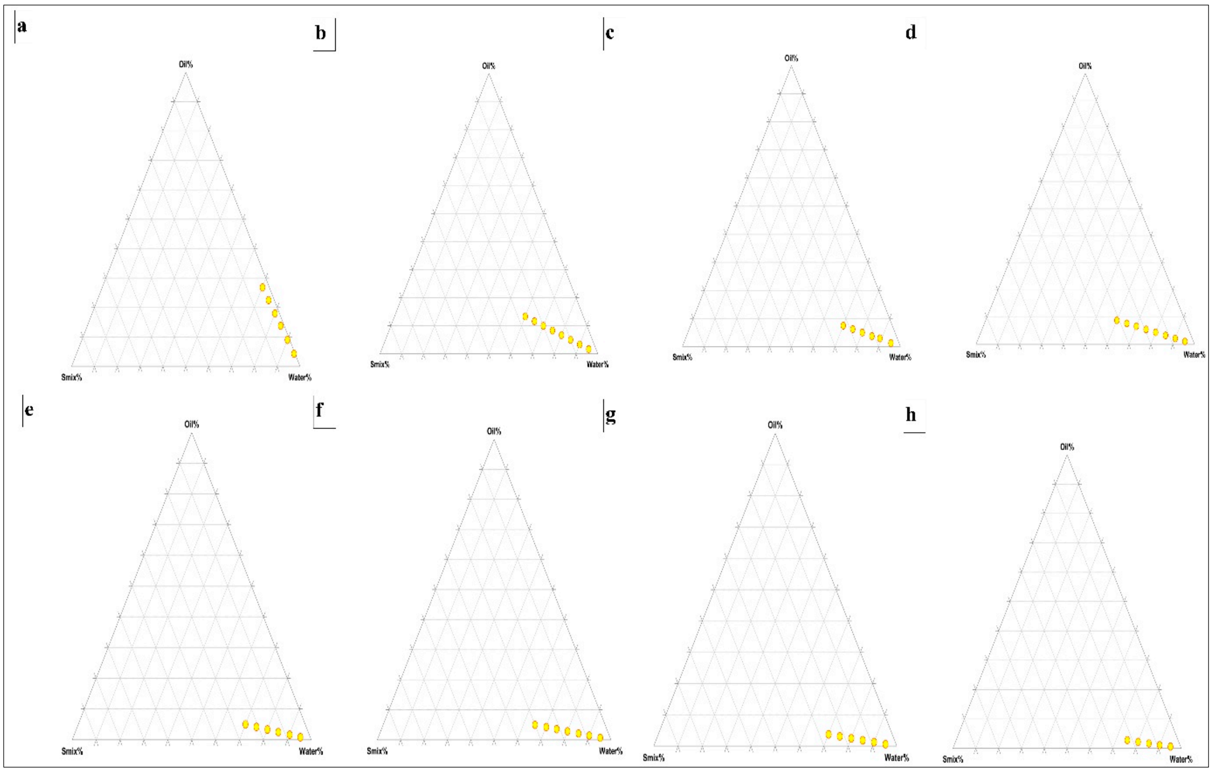

Figure S2: Pseudo-ternary phase diagrams were constructed using Capmul MCM-C8 as the oil phase, Transcutol-P as the surfactant, and Cremophor ELP as the co-surfactant. The squared region represents the O/W Microemulsion area observed at varying oil to Smix ratios: (a) 9:1, (b) 1:2, (c) 1:3, (d) 1:3.5, (e) 1:5, (f) 1:6, (g) 1:7, and (h) 1:8.
